# [3-(Iodo­acetamido)prop­yl]triphenyl­phospho­nium tetra­phenyl­borate

**DOI:** 10.1107/S160053681000142X

**Published:** 2010-01-16

**Authors:** Cameron Evans

**Affiliations:** a Department of Chemistry, University of Otago, PO Box 56, Dunedin, New Zealand

## Abstract

The title compound, C_23_H_24_INOP^+^·C_24_H_20_B^−^, was prepared by treatment of 3-amino­propyl triphenyl­phospho­nium bromide hydrogen bromide with *p*-nitro­phenyl iodo­acetate at 203 K. The asymmetric unit contains a single cation and anion, which are linked in the crystal by inter­molecular N—H⋯π and inversion-related *R*_2_^2^(14) C—H⋯O inter­actions, which combine to form chains of cations and anions along the *c* axis.

## Related literature

For the development and applications of mitochondrially targeted bio-active compounds, see Murphy & Smith (2007 ▶); Porteous *et al.* (2010 ▶). For the use of iodo­acetamides in labelling cysteine residues, see Baty *et al.* (2002 ▶); Kim *et al.* (2000 ▶); Ying *et al.* (2007 ▶). For the synthesis of amino­alkyl triphenyl­phospho­nium salts, see McAllister *et al.* (1980 ▶). For the synthesis of iodo­acetamides, see Trujillo *et al.* (1991 ▶). For related structures see Czerwinski (1986 ▶); Dubourg *et al.* (1986 ▶); Kerrigan *et al.*(1996 ▶); Lo *et al.*(2002 ▶). For a review of hydrogen bonding networks, see Bernstein *et al.*(1995 ▶).
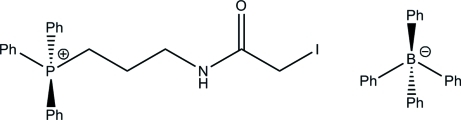


## Experimental

### 

#### Crystal data

C_23_H_24_INOP^+^·C_24_H_20_B^−^
*M* *_r_* = 807.51
Monoclinic, 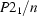

*a* = 14.552 (3) Å
*b* = 12.108 (2) Å
*c* = 21.966 (4) Å
β = 99.49 (3)°
*V* = 3817.3 (13) Å^3^
*Z* = 4
Mo *K*α radiation
μ = 0.92 mm^−1^
*T* = 89 K
0.22 × 0.20 × 0.2 0mm

#### Data collection

Bruker APEXII CCD area-detector diffractometer
Absorption correction: multi-scan (*SADABS*; Bruker, 2006 ▶) *T*_min_ = 0.661, *T*_max_ = 0.832
38267 measured reflections
9958 independent reflections
7291 reflections with *I* > 2σ(*I*)
*R*_int_ = 0.049

#### Refinement

*R*[*F* ^2^ > 2σ(*F* ^2^)] = 0.037
*wR*(*F* ^2^) = 0.103
*S* = 1.11
9958 reflections
469 parameters
H-atom parameters constrained
Δρ_max_ = 0.74 e Å^−3^
Δρ_min_ = −0.99 e Å^−3^

### 

Data collection: *APEX2* (Bruker, 2006 ▶); cell refinement: *APEX2* and *SAINT* (Bruker, 2006 ▶); data reduction: *SAINT* (Bruker, 2006 ▶); program(s) used to solve structure: *SIR97* (Altomare *et al.*, 1999 ▶); program(s) used to refine structure: *SHELXL97* (Sheldrick, 2008 ▶); molecular graphics: *ORTEP-3 for Windows* (Farrugia, 1997 ▶) and *Mercury* (Macrae *et al.*, 2006 ▶); software used to prepare material for publication: *WinGX* (Farrugia, 1999 ▶), *enCIFer* (Allen *et al.*, 2004 ▶) and *publCIF* (Westrip, 2010 ▶).

## Supplementary Material

Crystal structure: contains datablocks I, global. DOI: 10.1107/S160053681000142X/nc2174sup1.cif

Structure factors: contains datablocks I. DOI: 10.1107/S160053681000142X/nc2174Isup2.hkl

Additional supplementary materials:  crystallographic information; 3D view; checkCIF report

## Figures and Tables

**Table 1:** Hydrogen-bond geometry (Å, °) *Cg* is the centroid of the C61—C66 ring.

*D*—H⋯*A*	*D*—H	H⋯*A*	*D*⋯*A*	*D*—H⋯*A*
N1—H1⋯*Cg*^i^	0.86	2.56	3.382 (2)	160
C1—H1*B*⋯O1^ii^	0.97	2.48	3.270 (3)	139

Symmetry codes: (i) 
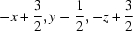
; (ii) 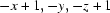.

**Fig. 1.**
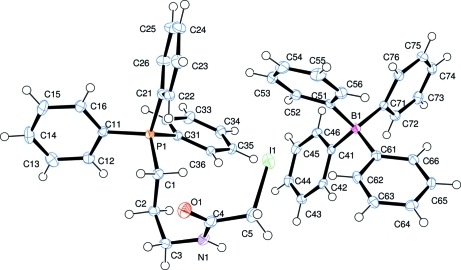
View of the two ions in the asymmetric unit showing the atom-labelling scheme. Ellipsoids are drawn at the 50% probability level with H atoms represented by circles of arbitrary size.

**Fig. 2.**
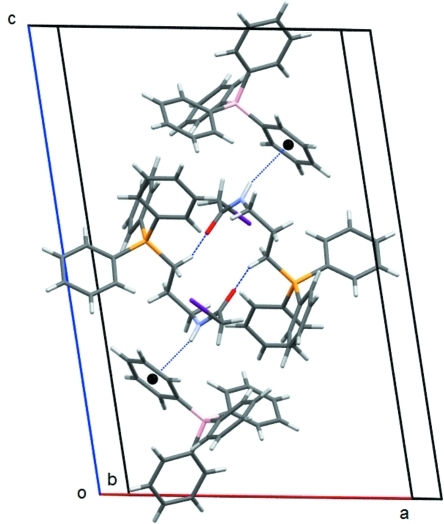
View along the c axis indicating the N···π and C—H···O R22(14) hydrogen bonding network in the crystal. Hydrogen bonds and the N—H···π interactions are drawn as dotted lines. Black spheres represent the centroids of the C61—C66 rings.

## supplementary crystallographic information

### Comment

One aspect of our research into mitochondrially targeted bio-active agents (Murphy and Smith, 2007) involves synthesis of a series of targeted iodoacetamides from aminoalkyl-triphenylphosphonium salts (Porteous *et al.*, 2010). The use of iodoacetamides in labelling of cysteine residues in proteins and peptides is well established (Ying *et al.*, 2007) allowing attachment of key markers such as fluorescein (Baty *et al.*, 2002) or biotin (Kim *et al.*, 2000). Given the widespread use of the iodoacetamide functionality it is surprising that there appears to be no structural data available for non-aryl iodoacetamides.

The title compound crystallizes with one cation and anion in the asymmetric unit (Fig. 1). The bond distances within the iodoacetamide functionality [C(5)—I(1) 2.172 (3) Å, N(1)—C(4) 1.344 (3)Å and C(4)—O(1) 1.233 (3) Å] are equivalent to those reported for 4-chloro-7-(iodoacetyl)amino-3-methoxy isocoumarin [2.139 (9) Å, 1.363 (13)Å and 1.209 (14) Å; Kerrigan *et al.*, 1996] and *N*-(ferrocenyl)iodoacetamide [2.152 (5) Å, 1.348 (6)Å and 1.234 (5) Å; Lo *et al.*, 2002] indicating that the presence of the triphenylphosphonium cation has a negligible effect. The C(1)—P(1) [1.810 (3) Å] and C(3)—N(1) [1.462 (3) Å] distances mirror those observed for both dimethylamino-3-propyl triphenylphosphonium chloride [1.802 (3)Å and 1.496 (9) Å; Dubourg *et al.*, 1986] and 2-aminoethyltriphenylphosphonium bromide hydrogen bromide [1.796 (5)Å and 1.512 (6) Å; Czerwinski, 1986].

The crystal packing is dominated by intermolecular N—H···π and C—H···O interactions (Fig. 2). The H(1)···CT and N(1)···CT distances [2.56Å and 3.382 (3) Å, where CT is the centroid of an adjacent C61—C66 ring on the tetraphenylborate anion] are indicative of a H-bonding interaction. In addition, there are inversion related C(1)—H(1B)···O(1) interactions [H(1B)···O(1) 2.48 Å, C(1)···O(1) 3.270 (3) Å] forming *R*^2^_2 _(14) ring motifs (Bernstein *et al.*, 1995). The combination of these two types of interactions form chains of cations and anions as viewed along the *c* axis.

### Experimental

The title compound was prepared from 3-aminopropyl triphenylphosphonium bromide hydrogen bromide (prepared using methods similar to McAllister *et al.*, 1980) using a modified literature procedure (Trujillo *et al.*, 1991). Triethylamine (0.43 mmol) was added to a dichloromethane solution (20 mL) of 3-aminopropyl triphenylphosphonium bromide hydrogen bromide (0.43 mmol), the solution cooled to -70°C and solid *p*-nitrophenyl iodoacetate (0.43 mmol) added in one portion. The solution was stirred at -70°C for 20 minutes and the solvent removed under vacuum. The solid residue was dissolved in acetone (5 mL), excess sodium tetraphenylborate (1 mmol) added and the solution stirred for 2 h at room temperature. Solvent was removed under vacuum, the compound redissolved in dichloromethane (2 mL) and precipitated by addition to diethylther (20 mL). Crystals were prepared by vapour diffusion of diethylether into an ethanolic solution of the compound at room temperature.

### Refinement

All H-atoms were positioned geometrically and refined using a riding model with d(C—H) = 0.93 Å, *U*_iso_=1.2*U*_eq_ (C) for aromatic and 0.97 Å, *U*_iso_ = 1.2*U*_eq_ (C) for CH_2_ and 0.86 Å, *U*_iso_ = 1.2*U*_eq_ (N) for the NH atom.

### Crystal data

**Table:**

C_23_H_24_INOP^+^·C_24_H_20_B^−^	*F*(000) = 1656
*M**_r_* = 807.51	*D*_x_ = 1.405 Mg m^−^^3^
Monoclinic, *P*2_1_/*n*	Mo *K*α radiation, λ = 0.71073 Å
Hall symbol: -P 2yn	Cell parameters from 6317 reflections
*a* = 14.552 (3) Å	θ = 2.5–28.7°
*b* = 12.108 (2) Å	µ = 0.92 mm^−^^1^
*c* = 21.966 (4) Å	*T* = 89 K
β = 99.49 (3)°	Prism, colourless
*V* = 3817.3 (13) Å^3^	0.22 × 0.2 × 0.2 mm
*Z* = 4	

### Data collection

**Table:**

Bruker APEXII CCD area-detector diffractometer	9958 independent reflections
Radiation source: fine-focus sealed tube	7291 reflections with *I* > 2σ(*I*)
graphite	*R*_int_ = 0.049
φ and ω scans	θ_max_ = 29.1°, θ_min_ = 2.3°
Absorption correction: multi-scan (*SADABS*; Bruker, 2006)	*h* = −13→19
*T*_min_ = 0.661, *T*_max_ = 0.832	*k* = −16→16
38267 measured reflections	*l* = −29→29

### Refinement

**Table:**

Refinement on *F*^2^	Primary atom site location: structure-invariant direct methods
Least-squares matrix: full	Secondary atom site location: difference Fourier map
*R*[*F*^2^ > 2σ(*F*^2^)] = 0.037	Hydrogen site location: inferred from neighbouring sites
*wR*(*F*^2^) = 0.103	H-atom parameters constrained
*S* = 1.11	*w* = 1/[σ^2^(*F*_o_^2^) + (0.0467*P*)^2^] where *P* = (*F*_o_^2^ + 2*F*_c_^2^)/3
9958 reflections	(Δ/σ)_max_ = 0.002
469 parameters	Δρ_max_ = 0.74 e Å^−^^3^
0 restraints	Δρ_min_ = −0.99 e Å^−^^3^

### Special details

**Table:**

Geometry. All e.s.d.'s (except the e.s.d. in the dihedral angle between two l.s. planes) are estimated using the full covariance matrix. The cell e.s.d.'s are taken into account individually in the estimation of e.s.d.'s in distances, angles and torsion angles; correlations between e.s.d.'s in cell parameters are only used when they are defined by crystal symmetry. An approximate (isotropic) treatment of cell e.s.d.'s is used for estimating e.s.d.'s involving l.s. planes.
Refinement. Refinement of *F*^2^ against ALL reflections. The weighted *R*-factor *wR* and goodness of fit *S* are based on *F*^2^, conventional *R*-factors *R* are based on *F*, with *F* set to zero for negative *F*^2^. The threshold expression of *F*^2^ > σ(*F*^2^) is used only for calculating *R*-factors(gt) *etc*. and is not relevant to the choice of reflections for refinement. *R*-factors based on *F*^2^ are statistically about twice as large as those based on *F*, and *R*- factors based on ALL data will be even larger.

### Fractional atomic coordinates and isotropic or equivalent isotropic displacement parameters (Å^2^)

**Table:**

	*x*	*y*	*z*	*U*_iso_*/*U*_eq_	
C1	0.64225 (17)	0.08981 (19)	0.51219 (11)	0.0171 (5)	
H1A	0.6231	0.158	0.5297	0.02*	
H1B	0.5865	0.0549	0.4902	0.02*	
C2	0.68361 (17)	0.01362 (19)	0.56566 (11)	0.0182 (5)	
H2A	0.6959	−0.0584	0.5494	0.022*	
H2B	0.7424	0.0439	0.5861	0.022*	
C3	0.61750 (18)	0.00073 (19)	0.61254 (11)	0.0204 (5)	
H3A	0.5628	−0.0399	0.5936	0.025*	
H3B	0.6482	−0.0423	0.6473	0.025*	
C4	0.51294 (18)	0.1611 (2)	0.60641 (11)	0.0204 (5)	
C5	0.49861 (18)	0.2755 (2)	0.63068 (12)	0.0240 (6)	
H5A	0.521	0.2783	0.6748	0.029*	
H5B	0.4328	0.2937	0.6235	0.029*	
C11	0.73474 (16)	0.00477 (19)	0.41211 (11)	0.0160 (5)	
C12	0.78439 (18)	−0.08572 (19)	0.44061 (12)	0.0203 (5)	
H12	0.8178	−0.0792	0.4804	0.024*	
C13	0.78336 (18)	−0.1849 (2)	0.40913 (13)	0.0262 (6)	
H13	0.815	−0.2457	0.4281	0.031*	
C14	0.73523 (18)	−0.1936 (2)	0.34938 (13)	0.0269 (6)	
H14	0.7342	−0.2608	0.3287	0.032*	
C15	0.6888 (2)	−0.1039 (2)	0.32005 (13)	0.0258 (6)	
H15	0.6585	−0.1098	0.2795	0.031*	
C16	0.68773 (18)	−0.0041 (2)	0.35196 (11)	0.0204 (5)	
H16	0.6555	0.0563	0.3329	0.024*	
C21	0.65711 (17)	0.22816 (18)	0.40569 (10)	0.0166 (5)	
C22	0.56359 (17)	0.25297 (19)	0.40603 (11)	0.0178 (5)	
H22	0.5315	0.2179	0.4338	0.021*	
C23	0.51862 (18)	0.3302 (2)	0.36477 (12)	0.0221 (6)	
H23	0.4562	0.3466	0.3648	0.026*	
C24	0.5667 (2)	0.3832 (2)	0.32344 (12)	0.0239 (6)	
H24	0.5361	0.4344	0.2957	0.029*	
C25	0.6600 (2)	0.3600 (2)	0.32343 (12)	0.0259 (6)	
H25	0.6921	0.3962	0.296	0.031*	
C26	0.70566 (18)	0.2824 (2)	0.36446 (11)	0.0221 (6)	
H26	0.7682	0.2667	0.3645	0.027*	
C31	0.82434 (17)	0.18234 (19)	0.49480 (10)	0.0153 (5)	
C32	0.90545 (17)	0.1740 (2)	0.46827 (11)	0.0187 (5)	
H32	0.906	0.1311	0.4332	0.022*	
C33	0.98484 (18)	0.2306 (2)	0.49516 (11)	0.0224 (6)	
H33	1.0388	0.227	0.4777	0.027*	
C34	0.98368 (18)	0.2925 (2)	0.54801 (12)	0.0221 (6)	
H34	1.0372	0.33	0.5658	0.027*	
C35	0.90392 (17)	0.29959 (19)	0.57498 (11)	0.0189 (5)	
H35	0.9045	0.3408	0.6108	0.023*	
C36	0.82383 (18)	0.24521 (18)	0.54849 (10)	0.0167 (5)	
H36	0.77	0.2502	0.566	0.02*	
N1	0.58821 (15)	0.10650 (16)	0.63510 (9)	0.0203 (5)	
H1	0.6202	0.1347	0.6678	0.024*	
O1	0.46005 (13)	0.12292 (14)	0.56173 (8)	0.0247 (4)	
P1	0.71663 (4)	0.12487 (5)	0.45667 (3)	0.01393 (14)	
I1	0.574759 (12)	0.392989 (13)	0.583303 (8)	0.02472 (7)	
C41	0.96560 (17)	0.56909 (19)	0.67978 (10)	0.0158 (5)	
C42	0.95432 (17)	0.48566 (19)	0.72216 (10)	0.0158 (5)	
H42	0.9033	0.4891	0.7426	0.019*	
C43	1.01627 (18)	0.39780 (19)	0.73499 (11)	0.0189 (5)	
H43	1.0056	0.3439	0.7632	0.023*	
C44	1.09355 (19)	0.3903 (2)	0.70599 (12)	0.0217 (6)	
H44	1.1347	0.3314	0.7143	0.026*	
C45	1.10912 (18)	0.4726 (2)	0.66406 (11)	0.0215 (6)	
H45	1.1613	0.4696	0.6447	0.026*	
C46	1.04555 (17)	0.5590 (2)	0.65170 (11)	0.0177 (5)	
H46	1.0564	0.6128	0.6235	0.021*	
C51	0.82725 (17)	0.64150 (19)	0.59114 (11)	0.0161 (5)	
C52	0.85473 (18)	0.5664 (2)	0.54902 (11)	0.0189 (5)	
H52	0.9147	0.5374	0.5572	0.023*	
C53	0.7963 (2)	0.5332 (2)	0.49554 (12)	0.0258 (6)	
H53	0.817	0.4818	0.4694	0.031*	
C54	0.7073 (2)	0.5763 (2)	0.48112 (12)	0.0265 (6)	
H54	0.6676	0.5533	0.4458	0.032*	
C55	0.67821 (19)	0.6545 (2)	0.52008 (12)	0.0269 (6)	
H55	0.6193	0.6858	0.5103	0.032*	
C56	0.73713 (18)	0.6860 (2)	0.57372 (11)	0.0220 (6)	
H56	0.7163	0.7386	0.5991	0.026*	
C61	0.81277 (16)	0.67687 (19)	0.70793 (10)	0.0153 (5)	
C62	0.74884 (17)	0.58898 (19)	0.71142 (11)	0.0176 (5)	
H62	0.7519	0.5276	0.6864	0.021*	
C63	0.68308 (18)	0.58957 (19)	0.74946 (11)	0.0190 (5)	
H63	0.6448	0.5284	0.7507	0.023*	
C64	0.67309 (18)	0.6812 (2)	0.78639 (11)	0.0199 (5)	
H64	0.6281	0.6823	0.8119	0.024*	
C65	0.73185 (17)	0.7703 (2)	0.78403 (11)	0.0195 (5)	
H65	0.7258	0.8328	0.8077	0.023*	
C66	0.80049 (17)	0.76719 (19)	0.74611 (11)	0.0177 (5)	
H66	0.8398	0.8278	0.7462	0.021*	
C71	0.94558 (16)	0.78910 (19)	0.66057 (10)	0.0152 (5)	
C72	1.03056 (18)	0.8086 (2)	0.69976 (11)	0.0200 (5)	
H72	1.0565	0.7519	0.7256	0.024*	
C73	1.07757 (19)	0.9091 (2)	0.70155 (12)	0.0233 (6)	
H73	1.1342	0.9178	0.7277	0.028*	
C74	1.04013 (19)	0.9965 (2)	0.66442 (11)	0.0220 (6)	
H74	1.071	1.0639	0.6656	0.026*	
C75	0.95639 (18)	0.9812 (2)	0.62579 (11)	0.0214 (5)	
H75	0.9306	1.0386	0.6005	0.026*	
C76	0.91000 (18)	0.87998 (18)	0.62445 (11)	0.0179 (5)	
H76	0.853	0.8724	0.5985	0.021*	
B1	0.8892 (2)	0.6704 (2)	0.66017 (12)	0.0159 (6)	

### Atomic displacement parameters (Å^2^)

**Table:**

	*U*^11^	*U*^22^	*U*^33^	*U*^12^	*U*^13^	*U*^23^
C1	0.0157 (12)	0.0186 (13)	0.0157 (12)	−0.0002 (9)	−0.0007 (10)	−0.0002 (9)
C2	0.0203 (13)	0.0175 (12)	0.0160 (12)	−0.0005 (10)	0.0004 (10)	0.0020 (10)
C3	0.0259 (14)	0.0173 (13)	0.0174 (12)	−0.0036 (10)	0.0016 (11)	0.0040 (10)
C4	0.0197 (13)	0.0252 (14)	0.0178 (12)	−0.0049 (10)	0.0078 (11)	−0.0010 (11)
C5	0.0222 (14)	0.0271 (14)	0.0238 (14)	0.0007 (11)	0.0069 (11)	−0.0051 (11)
C11	0.0156 (12)	0.0179 (12)	0.0149 (11)	−0.0014 (9)	0.0038 (10)	−0.0006 (9)
C12	0.0192 (13)	0.0203 (13)	0.0202 (13)	0.0029 (10)	−0.0006 (11)	0.0004 (10)
C13	0.0201 (14)	0.0210 (14)	0.0378 (16)	0.0034 (10)	0.0055 (12)	0.0010 (12)
C14	0.0235 (14)	0.0225 (14)	0.0372 (16)	−0.0044 (11)	0.0125 (13)	−0.0142 (12)
C15	0.0246 (15)	0.0299 (15)	0.0218 (14)	−0.0054 (11)	0.0006 (12)	−0.0099 (11)
C16	0.0211 (13)	0.0207 (13)	0.0182 (12)	−0.0026 (10)	−0.0004 (11)	−0.0011 (10)
C21	0.0187 (13)	0.0134 (12)	0.0158 (12)	−0.0001 (9)	−0.0027 (10)	−0.0008 (9)
C22	0.0204 (13)	0.0149 (12)	0.0170 (12)	−0.0002 (9)	−0.0001 (10)	−0.0002 (10)
C23	0.0183 (13)	0.0213 (14)	0.0241 (13)	0.0047 (10)	−0.0035 (11)	−0.0001 (11)
C24	0.0295 (15)	0.0178 (13)	0.0223 (14)	0.0035 (10)	−0.0014 (12)	0.0050 (10)
C25	0.0284 (15)	0.0243 (14)	0.0246 (14)	−0.0009 (11)	0.0031 (12)	0.0097 (11)
C26	0.0187 (13)	0.0259 (14)	0.0215 (13)	0.0001 (11)	0.0026 (11)	0.0054 (11)
C31	0.0172 (12)	0.0144 (12)	0.0129 (11)	0.0015 (9)	−0.0019 (10)	0.0008 (9)
C32	0.0196 (13)	0.0219 (13)	0.0139 (12)	0.0003 (10)	0.0007 (10)	−0.0034 (10)
C33	0.0170 (13)	0.0292 (15)	0.0208 (13)	−0.0016 (11)	0.0022 (11)	−0.0036 (11)
C34	0.0192 (13)	0.0223 (13)	0.0229 (13)	−0.0034 (10)	−0.0026 (11)	−0.0032 (11)
C35	0.0256 (14)	0.0168 (13)	0.0134 (12)	−0.0015 (10)	0.0005 (11)	−0.0028 (9)
C36	0.0211 (13)	0.0142 (12)	0.0146 (11)	0.0015 (9)	0.0021 (10)	0.0001 (9)
N1	0.0238 (12)	0.0224 (11)	0.0138 (10)	−0.0016 (9)	0.0007 (9)	−0.0022 (8)
O1	0.0191 (10)	0.0288 (10)	0.0253 (10)	−0.0038 (7)	0.0005 (8)	−0.0048 (8)
P1	0.0146 (3)	0.0143 (3)	0.0118 (3)	0.0008 (2)	−0.0009 (2)	0.0000 (2)
I1	0.02367 (10)	0.01857 (10)	0.03027 (11)	0.00050 (7)	−0.00038 (8)	−0.00280 (7)
C41	0.0184 (12)	0.0158 (12)	0.0110 (11)	−0.0013 (9)	−0.0041 (10)	−0.0040 (9)
C42	0.0163 (12)	0.0184 (12)	0.0120 (11)	0.0009 (9)	0.0001 (10)	−0.0028 (9)
C43	0.0236 (14)	0.0172 (13)	0.0150 (12)	0.0009 (10)	0.0007 (11)	0.0006 (10)
C44	0.0246 (14)	0.0191 (13)	0.0188 (13)	0.0078 (10)	−0.0042 (11)	−0.0028 (10)
C45	0.0177 (13)	0.0284 (14)	0.0176 (12)	0.0043 (11)	0.0007 (11)	−0.0063 (11)
C46	0.0197 (13)	0.0193 (12)	0.0133 (12)	0.0004 (10)	0.0000 (10)	0.0003 (10)
C51	0.0213 (13)	0.0109 (11)	0.0158 (12)	−0.0017 (9)	0.0019 (10)	0.0024 (9)
C52	0.0200 (13)	0.0173 (12)	0.0187 (13)	0.0000 (10)	0.0014 (11)	0.0025 (10)
C53	0.0397 (17)	0.0193 (14)	0.0163 (13)	−0.0018 (11)	−0.0014 (12)	−0.0023 (10)
C54	0.0310 (16)	0.0281 (15)	0.0166 (13)	−0.0054 (12)	−0.0074 (12)	0.0011 (11)
C55	0.0217 (14)	0.0336 (16)	0.0223 (14)	0.0021 (12)	−0.0057 (12)	0.0061 (12)
C56	0.0248 (14)	0.0206 (13)	0.0192 (13)	0.0013 (10)	−0.0004 (11)	0.0005 (10)
C61	0.0144 (12)	0.0152 (12)	0.0146 (11)	0.0036 (9)	−0.0028 (10)	0.0021 (9)
C62	0.0177 (12)	0.0133 (12)	0.0199 (13)	0.0025 (9)	−0.0027 (10)	−0.0013 (9)
C63	0.0192 (13)	0.0152 (13)	0.0220 (13)	0.0022 (9)	0.0014 (11)	0.0013 (10)
C64	0.0183 (13)	0.0242 (14)	0.0172 (12)	0.0032 (10)	0.0031 (10)	0.0036 (10)
C65	0.0260 (14)	0.0156 (13)	0.0168 (12)	0.0031 (10)	0.0029 (11)	−0.0036 (10)
C66	0.0186 (13)	0.0150 (12)	0.0182 (12)	−0.0014 (9)	−0.0008 (10)	0.0012 (10)
C71	0.0178 (12)	0.0159 (12)	0.0119 (11)	0.0013 (9)	0.0028 (10)	−0.0020 (9)
C72	0.0237 (14)	0.0184 (13)	0.0161 (12)	−0.0002 (10)	−0.0019 (11)	−0.0006 (10)
C73	0.0238 (14)	0.0235 (14)	0.0213 (14)	−0.0034 (10)	0.0003 (12)	−0.0073 (11)
C74	0.0285 (15)	0.0153 (13)	0.0243 (14)	−0.0033 (10)	0.0103 (12)	−0.0033 (10)
C75	0.0278 (15)	0.0164 (13)	0.0204 (13)	0.0044 (10)	0.0051 (11)	0.0044 (10)
C76	0.0176 (13)	0.0172 (13)	0.0185 (12)	0.0040 (9)	0.0018 (10)	−0.0002 (10)
B1	0.0187 (14)	0.0131 (13)	0.0141 (13)	0.0007 (10)	−0.0027 (11)	0.0010 (10)

### Geometric parameters (Å, °)

**Table:**

C1—C2	1.536 (3)	C36—H36	0.93
C1—P1	1.810 (3)	N1—H1	0.86
C1—H1A	0.97	C41—C42	1.402 (3)
C1—H1B	0.97	C41—C46	1.409 (3)
C2—C3	1.529 (3)	C41—B1	1.664 (4)
C2—H2A	0.97	C42—C43	1.393 (3)
C2—H2B	0.97	C42—H42	0.93
C3—N1	1.462 (3)	C43—C44	1.384 (4)
C3—H3A	0.97	C43—H43	0.93
C3—H3B	0.97	C44—C45	1.400 (4)
C4—O1	1.233 (3)	C44—H44	0.93
C4—N1	1.344 (3)	C45—C46	1.394 (3)
C4—C5	1.511 (3)	C45—H45	0.93
C5—I1	2.172 (3)	C46—H46	0.93
C5—H5A	0.97	C51—C52	1.402 (4)
C5—H5B	0.97	C51—C56	1.411 (3)
C11—C16	1.388 (3)	C51—B1	1.668 (3)
C11—C12	1.402 (3)	C52—C53	1.391 (3)
C11—P1	1.796 (2)	C52—H52	0.93
C12—C13	1.384 (3)	C53—C54	1.383 (4)
C12—H12	0.93	C53—H53	0.93
C13—C14	1.386 (4)	C54—C55	1.389 (4)
C13—H13	0.93	C54—H54	0.93
C14—C15	1.382 (4)	C55—C56	1.391 (3)
C14—H14	0.93	C55—H55	0.93
C15—C16	1.398 (3)	C56—H56	0.93
C15—H15	0.93	C61—C66	1.407 (3)
C16—H16	0.93	C61—C62	1.424 (3)
C21—C22	1.395 (3)	C61—B1	1.652 (4)
C21—C26	1.401 (3)	C62—C63	1.370 (4)
C21—P1	1.802 (2)	C62—H62	0.93
C22—C23	1.389 (3)	C63—C64	1.396 (3)
C22—H22	0.93	C63—H63	0.93
C23—C24	1.391 (4)	C64—C65	1.383 (3)
C23—H23	0.93	C64—H64	0.93
C24—C25	1.387 (4)	C65—C66	1.403 (3)
C24—H24	0.93	C65—H65	0.93
C25—C26	1.392 (3)	C66—H66	0.93
C25—H25	0.93	C71—C76	1.405 (3)
C26—H26	0.93	C71—C72	1.405 (3)
C31—C32	1.404 (3)	C71—B1	1.654 (4)
C31—C36	1.405 (3)	C72—C73	1.394 (3)
C31—P1	1.791 (2)	C72—H72	0.93
C32—C33	1.389 (3)	C73—C74	1.391 (4)
C32—H32	0.93	C73—H73	0.93
C33—C34	1.384 (3)	C74—C75	1.378 (4)
C33—H33	0.93	C74—H74	0.93
C34—C35	1.390 (4)	C75—C76	1.397 (3)
C34—H34	0.93	C75—H75	0.93
C35—C36	1.381 (3)	C76—H76	0.93
C35—H35	0.93		
			
C2—C1—P1	116.98 (17)	C31—P1—C11	111.93 (11)
C2—C1—H1A	108.1	C31—P1—C21	108.34 (11)
P1—C1—H1A	108.1	C11—P1—C21	108.77 (11)
C2—C1—H1B	108.1	C31—P1—C1	110.52 (11)
P1—C1—H1B	108.1	C11—P1—C1	109.58 (11)
H1A—C1—H1B	107.3	C21—P1—C1	107.58 (11)
C3—C2—C1	111.4 (2)	C42—C41—C46	115.1 (2)
C3—C2—H2A	109.3	C42—C41—B1	123.8 (2)
C1—C2—H2A	109.3	C46—C41—B1	121.0 (2)
C3—C2—H2B	109.3	C43—C42—C41	122.8 (2)
C1—C2—H2B	109.3	C43—C42—H42	118.6
H2A—C2—H2B	108	C41—C42—H42	118.6
N1—C3—C2	112.95 (19)	C44—C43—C42	120.3 (2)
N1—C3—H3A	109	C44—C43—H43	119.8
C2—C3—H3A	109	C42—C43—H43	119.8
N1—C3—H3B	109	C43—C44—C45	119.2 (2)
C2—C3—H3B	109	C43—C44—H44	120.4
H3A—C3—H3B	107.8	C45—C44—H44	120.4
O1—C4—N1	122.9 (2)	C46—C45—C44	119.2 (2)
O1—C4—C5	121.3 (2)	C46—C45—H45	120.4
N1—C4—C5	115.8 (2)	C44—C45—H45	120.4
C4—C5—I1	108.70 (16)	C45—C46—C41	123.3 (2)
C4—C5—H5A	110	C45—C46—H46	118.3
I1—C5—H5A	110	C41—C46—H46	118.3
C4—C5—H5B	110	C52—C51—C56	115.0 (2)
I1—C5—H5B	110	C52—C51—B1	124.5 (2)
H5A—C5—H5B	108.3	C56—C51—B1	120.3 (2)
C16—C11—C12	120.1 (2)	C53—C52—C51	122.9 (2)
C16—C11—P1	119.20 (19)	C53—C52—H52	118.5
C12—C11—P1	120.03 (18)	C51—C52—H52	118.5
C13—C12—C11	119.5 (2)	C54—C53—C52	120.2 (2)
C13—C12—H12	120.2	C54—C53—H53	119.9
C11—C12—H12	120.2	C52—C53—H53	119.9
C12—C13—C14	120.1 (2)	C53—C54—C55	119.1 (2)
C12—C13—H13	120	C53—C54—H54	120.5
C14—C13—H13	120	C55—C54—H54	120.5
C15—C14—C13	120.9 (2)	C54—C55—C56	120.1 (3)
C15—C14—H14	119.5	C54—C55—H55	120
C13—C14—H14	119.5	C56—C55—H55	120
C14—C15—C16	119.4 (2)	C55—C56—C51	122.7 (2)
C14—C15—H15	120.3	C55—C56—H56	118.7
C16—C15—H15	120.3	C51—C56—H56	118.7
C11—C16—C15	120.0 (2)	C66—C61—C62	113.6 (2)
C11—C16—H16	120	C66—C61—B1	125.5 (2)
C15—C16—H16	120	C62—C61—B1	120.9 (2)
C22—C21—C26	119.9 (2)	C63—C62—C61	123.9 (2)
C22—C21—P1	121.02 (18)	C63—C62—H62	118.1
C26—C21—P1	119.03 (19)	C61—C62—H62	118.1
C23—C22—C21	119.8 (2)	C62—C63—C64	120.6 (2)
C23—C22—H22	120.1	C62—C63—H63	119.7
C21—C22—H22	120.1	C64—C63—H63	119.7
C22—C23—C24	120.2 (2)	C65—C64—C63	118.3 (2)
C22—C23—H23	119.9	C65—C64—H64	120.9
C24—C23—H23	119.9	C63—C64—H64	120.9
C25—C24—C23	120.3 (2)	C64—C65—C66	120.4 (2)
C25—C24—H24	119.8	C64—C65—H65	119.8
C23—C24—H24	119.8	C66—C65—H65	119.8
C24—C25—C26	119.9 (3)	C65—C66—C61	123.2 (2)
C24—C25—H25	120	C65—C66—H66	118.4
C26—C25—H25	120	C61—C66—H66	118.4
C25—C26—C21	119.8 (2)	C76—C71—C72	114.8 (2)
C25—C26—H26	120.1	C76—C71—B1	122.8 (2)
C21—C26—H26	120.1	C72—C71—B1	122.3 (2)
C32—C31—C36	120.6 (2)	C73—C72—C71	122.8 (2)
C32—C31—P1	120.57 (18)	C73—C72—H72	118.6
C36—C31—P1	118.53 (19)	C71—C72—H72	118.6
C33—C32—C31	119.0 (2)	C74—C73—C72	120.3 (2)
C33—C32—H32	120.5	C74—C73—H73	119.8
C31—C32—H32	120.5	C72—C73—H73	119.8
C34—C33—C32	120.0 (3)	C75—C74—C73	118.7 (2)
C34—C33—H33	120	C75—C74—H74	120.7
C32—C33—H33	120	C73—C74—H74	120.7
C33—C34—C35	121.1 (2)	C74—C75—C76	120.4 (2)
C33—C34—H34	119.4	C74—C75—H75	119.8
C35—C34—H34	119.4	C76—C75—H75	119.8
C36—C35—C34	119.8 (2)	C75—C76—C71	122.9 (2)
C36—C35—H35	120.1	C75—C76—H76	118.5
C34—C35—H35	120.1	C71—C76—H76	118.5
C35—C36—C31	119.4 (2)	C61—B1—C71	109.94 (19)
C35—C36—H36	120.3	C61—B1—C41	111.19 (19)
C31—C36—H36	120.3	C71—B1—C41	109.2 (2)
C4—N1—C3	122.2 (2)	C61—B1—C51	105.54 (19)
C4—N1—H1	118.9	C71—B1—C51	112.36 (19)
C3—N1—H1	118.9	C41—B1—C51	108.55 (19)
			
P1—C1—C2—C3	173.97 (16)	C41—C42—C43—C44	0.6 (4)
C1—C2—C3—N1	−53.6 (3)	C42—C43—C44—C45	0.6 (4)
O1—C4—C5—I1	−89.9 (3)	C43—C44—C45—C46	−1.1 (4)
N1—C4—C5—I1	87.8 (2)	C44—C45—C46—C41	0.5 (4)
C16—C11—C12—C13	−2.3 (4)	C42—C41—C46—C45	0.6 (3)
P1—C11—C12—C13	168.1 (2)	B1—C41—C46—C45	−176.5 (2)
C11—C12—C13—C14	1.4 (4)	C56—C51—C52—C53	−3.3 (4)
C12—C13—C14—C15	0.8 (4)	B1—C51—C52—C53	171.2 (2)
C13—C14—C15—C16	−2.2 (4)	C51—C52—C53—C54	1.5 (4)
C12—C11—C16—C15	0.9 (4)	C52—C53—C54—C55	1.2 (4)
P1—C11—C16—C15	−169.6 (2)	C53—C54—C55—C56	−1.9 (4)
C14—C15—C16—C11	1.4 (4)	C54—C55—C56—C51	−0.1 (4)
C26—C21—C22—C23	1.0 (3)	C52—C51—C56—C55	2.6 (4)
P1—C21—C22—C23	−177.79 (18)	B1—C51—C56—C55	−172.1 (2)
C21—C22—C23—C24	−0.4 (4)	C66—C61—C62—C63	−1.9 (3)
C22—C23—C24—C25	−0.5 (4)	B1—C61—C62—C63	−179.2 (2)
C23—C24—C25—C26	0.7 (4)	C61—C62—C63—C64	2.3 (4)
C24—C25—C26—C21	0.0 (4)	C62—C63—C64—C65	−0.7 (4)
C22—C21—C26—C25	−0.8 (4)	C63—C64—C65—C66	−1.0 (4)
P1—C21—C26—C25	178.01 (19)	C64—C65—C66—C61	1.4 (4)
C36—C31—C32—C33	−1.3 (3)	C62—C61—C66—C65	0.0 (3)
P1—C31—C32—C33	172.33 (19)	B1—C61—C66—C65	177.3 (2)
C31—C32—C33—C34	1.2 (4)	C76—C71—C72—C73	−1.8 (4)
C32—C33—C34—C35	−0.2 (4)	B1—C71—C72—C73	−178.4 (2)
C33—C34—C35—C36	−0.8 (4)	C71—C72—C73—C74	1.2 (4)
C34—C35—C36—C31	0.7 (3)	C72—C73—C74—C75	−0.4 (4)
C32—C31—C36—C35	0.4 (3)	C73—C74—C75—C76	0.4 (4)
P1—C31—C36—C35	−173.39 (17)	C74—C75—C76—C71	−1.2 (4)
O1—C4—N1—C3	4.9 (4)	C72—C71—C76—C75	1.8 (4)
C5—C4—N1—C3	−172.7 (2)	B1—C71—C76—C75	178.4 (2)
C2—C3—N1—C4	88.6 (3)	C66—C61—B1—C71	−3.3 (3)
C32—C31—P1—C11	31.1 (2)	C62—C61—B1—C71	173.7 (2)
C36—C31—P1—C11	−155.11 (18)	C66—C61—B1—C41	117.8 (2)
C32—C31—P1—C21	−88.8 (2)	C62—C61—B1—C41	−65.2 (3)
C36—C31—P1—C21	85.0 (2)	C66—C61—B1—C51	−124.7 (2)
C32—C31—P1—C1	153.57 (19)	C62—C61—B1—C51	52.3 (3)
C36—C31—P1—C1	−32.7 (2)	C76—C71—B1—C61	−83.7 (3)
C16—C11—P1—C31	−132.7 (2)	C72—C71—B1—C61	92.7 (3)
C12—C11—P1—C31	56.9 (2)	C76—C71—B1—C41	154.0 (2)
C16—C11—P1—C21	−13.0 (2)	C72—C71—B1—C41	−29.6 (3)
C12—C11—P1—C21	176.6 (2)	C76—C71—B1—C51	33.5 (3)
C16—C11—P1—C1	104.4 (2)	C72—C71—B1—C51	−150.1 (2)
C12—C11—P1—C1	−66.1 (2)	C42—C41—B1—C61	13.2 (3)
C22—C21—P1—C31	−130.73 (19)	C46—C41—B1—C61	−169.9 (2)
C26—C21—P1—C31	50.4 (2)	C42—C41—B1—C71	134.7 (2)
C22—C21—P1—C11	107.4 (2)	C46—C41—B1—C71	−48.4 (3)
C26—C21—P1—C11	−71.4 (2)	C42—C41—B1—C51	−102.5 (2)
C22—C21—P1—C1	−11.2 (2)	C46—C41—B1—C51	74.4 (3)
C26—C21—P1—C1	169.94 (19)	C52—C51—B1—C61	−138.3 (2)
C2—C1—P1—C31	−54.4 (2)	C56—C51—B1—C61	35.9 (3)
C2—C1—P1—C11	69.4 (2)	C52—C51—B1—C71	101.9 (3)
C2—C1—P1—C21	−172.53 (17)	C56—C51—B1—C71	−83.9 (3)
C46—C41—C42—C43	−1.2 (3)	C52—C51—B1—C41	−19.1 (3)
B1—C41—C42—C43	175.9 (2)	C56—C51—B1—C41	155.2 (2)

### Hydrogen-bond geometry (Å, °)

**Table:**

CT01 is the centroid of the C61—C66 ring.

**Table:**

*D*—H···*A*	*D*—H	H···*A*	*D*···*A*	*D*—H···*A*
N1—H1···CT01^i^	0.86	2.56	3.382 (2)	160
C1—H1B···O1^ii^	0.97	2.48	3.270 (3)	139

Symmetry codes: (i) −*x*+3/2, *y*−1/2, −*z*+3/2; (ii) −*x*+1, −*y*, −*z*+1.
